# Adaptive Messung der Sprachverständlichkeitsschwelle im Störschall mit einem verkürzten Freiburger Einsilbertest im Vergleich zum Oldenburger Satztest

**DOI:** 10.1055/a-2547-6600

**Published:** 2025-05-05

**Authors:** Gero Faustmann, Rainer Schönweiler, Jan Löhler

**Affiliations:** 1 9191 Klinik für Hals- Nasen- und Ohrenheilkunde, Universität zu Lübeck, Lubeck, Germany; 2 Universitätsklinikum Schleswig-Holstein, Phoniatrie, Lübeck, Germany; 3 HNO-Praxis Bad Bramstedt, Wissenschaftlichen Instituts für angewandte HNO-Heilkunde, Bad Bramstedt, Germany

**Keywords:** Sprachaudiometrie, Freiburger Einsilbertest, Oldenburger Satztest, Sprachaudiometrie im Störgeräusch, adaptive Messung, Freiburger Einsilbertest in verkürzter Version, Speech audiometry, Freiburg mono-syllabic speech-test, Oldenburg sentence test, speech discrimination test in noise, Freiburg mono-syllabic speech-test in shortened version

## Abstract

**Hintergrund:**

Der Freiburger Sprachtest basierte bislang auf einer absolut prozentualen, nicht adaptiven Messweise zur Bestimmung der Sprachverständlichkeit. Eine adaptive Pegelsteuerung ermöglicht jedoch die präzise und effiziente Bestimmung der 50%-Sprachverständlichkeitsschwelle (50%-SVS), indem der Sprachschallpegel individuell angepasst wird. In einer Vergleichsstudie führten Memmeler et al. bereits den Freiburger Einsilbertest mittels adaptiver Messmethode im Störschall (aFBE-S) erfolgreich durch. Zudem konnten theoretische Erkenntnisse für eine verkürzte Version des aFBE-S aus den bereits gewonnenen Daten berechnet werden. Ziel dieser Studie war es, die praktische Anwendbarkeit des verkürzten aFBE-S mit 3, 4 und 5 Testlisten zu prüfen und mit dem adaptiven Oldenburger Satztest (OLSA-S) zu vergleichen.

**Methoden:**

40 otologisch gesunde Probanden (18–30 Jahre) nahmen teil. Sie absolvierten den aFBE-S mit 3, 4 und 5 Testlisten sowie den OLSA-S in zufälliger Reihenfolge. Untersucht wurden die gemittelten Signal-Rausch-Abstände (SNR), der Zeitaufwand sowie der Einfluss von Reihenfolge und Geschlecht.

**Ergebnisse:**

Es gab keine signifikanten Unterschiede zwischen den SNR-Werten des verkürzten aFBE-S mit 3, 4 und 5 Testlisten und denen des OLSA-S. Auch zwischen den Ergebnissen des aFBE-S mit 3, 4 und 5 Testlisten zeigten sich keine signifikanten Differenzen. Die Reihenfolge und das Geschlecht beeinflussten die Ergebnisse nicht. Die Durchführung des verkürzten aFBE-S war signifikant schneller als die des OLSA-S und wies eine geringere Streuung auf.

**Schlussfolgerung:**

Der verkürzte aFBE-S mit 3, 4 und 5 Testlisten liefert im Vergleich zum OLSA-S vergleichbare SNR-Werte bei kürzerer Testzeit. Der aFBE-S mit 3 Testlisten erweist sich als ausreichend und kann für die klinische Praxis empfohlen werden.

## Abstract (en)

**Background:**

The Freiburg speech test is based on an absolute percentage, non-adaptive measurement method for determining speech intelligibility. Adaptive level control makes it possible to precisely and efficiently determine the 50 % speech intelligibility threshold (50 %-SVS) by individually adjusting the speech sound level. Memmeler et al. already successfully conducted the Freiburg monosyllabic test adaptively in noise (aFBE-S) and developed a shortened version based on existing data. The aim of this study was to test the practical applicability of the shortened aFBE-S with 3, 4 and 5 test lists and to compare it with the adaptive Oldenburg sentence test (OLSA-S).

**Methods:**

40 otologically healthy subjects (18–30 years) took part. They completed the aFBE-S with 3, 4 and 5 test lists and the OLSA-S in random order. The averaged signal-to-noise ratios (SNR), the time required and the influence of gender and order of measurements were investigated.

**Results:**

There were no significant differences between the SNR values of the shortened aFBE-S with 3, 4 and 5 test lists and those of the OLSA-S. There were also no significant differences between the results of the aFBE-S with 3, 4 and 5 test lists. The order and gender did not influence the results. The performance of the shortened aFBE-S was significantly faster than that of the OLSA-S and showed less variability.

**Conclusion:**

Compared to the OLSA-S, the shortened aFBE-S with 3, 4 and 5 test lists provides comparable SNR values in a shorter test time. The aFBE-S with 3 test lists proves to be sufficient and can be recommended for clinical practice.

## Hintergrund

Der Freiburger Sprachverständlichkeitstest stellt seit seiner Einführung vor über 70 Jahren ein bis heute sehr weit verbreitetes sprachaudiometrisches Verfahren im deutschsprachigen Raum dar. Neben der Differenzialdiagnostik und Begutachtung von Hörschäden kommt er insbesondere bei der Indikationsstellung und Anpassung von Hörhilfen nach der Hilfsmittel-Richtlinie (HilfsM-RL) sowie bei der Indikationsstellung und Nachsorge im Rahmen einer Cochlea-Implantation zur Anwendung. Er gliedert sich in einen Bereich, in dem die 50%-Verstehensquote mehrsilbiger Zahlen (Freiburger Zahlentest), und einen zweiten Bereich, in dem die absolute Verstehensquote einsilbiger Testwörter ermittelt wird (Freiburger Einsilbertest). Gemäß der HilfsM-RL ist für die beidohrige Hörgeräteversorgung neben einem tonaudiometrisch ermittelten Hörverlust von mindestens 30dB in mindestens 2 Testfrequenzen zwischen 0,5 und 4kHz auch eine Verstehensquote von ≤80% beim Freiburger Einsilbertest auf dem jeweils besser hörenden Ohr vorgegeben. Die Überprüfung mittels Freiburger Einsibertests lässt sich dabei sowohl ohne als auch mit Störschall für die „Überprüfung des Hörhilfenversorgungsergebnisses“ anwenden 1 2 3 4 .

Wesentliche Vorteile des Freiburger Einsilbertests (FBE) sind neben der unkomplizierten und raschen Testdurchführung auch die langjährigen Erfahrungen mit diesem Verfahren, die den Erhalt von validen und normierten Ergebnissen erlauben. Hinzu kommen die preiswerte Anschaffung und kostengünstige Durchführung. Zudem wurde auch eine Normkurve zum Verstehen von Einsilbern im Störschall ermittelt (FBE-S), was insbesondere für die Hörgeräteversorgung nach der HilfsM-RL von Bedeutung ist 5 6 7 .

Im Gegensatz zum Oldenburger Satztest basiert der Freiburger Sprachtest primär nicht auf einer adaptiven Messweise zur Beschreibung des Sprachverstehens. Für die Ermittlung der 50%-Sprachverständlichkeitsschwelle (50%-SVS) werden so viele Punkte der Diskriminationskurve gemessen, dass deren Wendepunkt bestimmt werden kann. Mit einem adaptiven Paradigma wird dieses Ziel i.d.R. schneller erreicht 1 .

Um zu überprüfen, ob sich auch der FBE im Störschall adaptiv verwenden lässt (aFBE-S), führten *Memmeler et al.* (2018) eine Vergleichsstudie durch, in der der OLSA-S und der aFBE-S mit jeweils demselben Algorithmus bei adaptiver Messweise verglichen wurden. Es stellte sich heraus, dass der aFBE-S, entgegen der Erwartung, für die adaptive Testdurchführung ungeeignet zu sein, sehr gut adaptiv durchgeführt werden kann und dass mit dem aFBE-S grundsätzlich zum OLSA-S vergleichbare Ergebnisse erzielt werden können 5 8 .

Gleichwohl konstatierten *Memmeler et al.* auch, dass die Durchführung des aFBE-S einen erheblich höheren Zeitaufwand erfordere, da primär 7,5 Testlisten verwendet wurden, um die gleiche Anzahl an Testitems wie beim OLSA-S zu verwenden. Ferner kritisierten *Holube et al.* bei dieser Untersuchung die fehlende Berücksichtigung der hohen Pegelfluktuationen der Einsilber sowie der verminderten Verdeckungseigenschaften des CCITT-Rauschens gegenüber dem sprachsimulierenden Rauschen des OLSA-S. Sie empfahlen eine Anpassung der ermittelten Signal-Rausch-Abstände des aFBE-S um –5,9dB, basierend auf dem äquivalenten Dauerschalldruckpegel der Sprache (L _eq_ ), um Unterschiede in der Trennschärfe der Tests aufzuheben 9 10 .

Diese Empfehlung berücksichtigten *Memmeler et al.* in einer nachfolgenden Studie, in der aus den bereits generierten Daten theoretisch ermittelt wurde, ob der aFBE-S in verkürzter Version mithilfe von 5, 4 oder 3 Testlisten durchgeführt werden könne. Hierbei zeigte sich, dass der aFBE-S theoretisch mit 5, 4 und 3 Testlisten statistisch vergleichbare Ergebnisse wie der OLSA-S liefern könnte. Gleichzeitig ergaben die Berechnungen, dass der aFBE-S in verkürzter Version mit 5, 4 und 3 Testlisten signifikant schneller durchzuführen wäre als der OLSA-S 11 . Hierauf aufbauend sollten in dieser Studie die Übertragung der adaptiven Pegeländerung auf den FBE in verkürzter Version untersucht und die zuvor theoretisch ermittelten Ergebnisse praktisch überprüft werden.

## Methoden

Für die Studie wurden 40 Probanden im Altersspektrum zwischen 18 und 30 Jahren (Durchschnittsalter 24,5 Jahre) rekrutiert. Die Einschlusskriterien zur Teilnahme umfassten neben dem o.g. Alter Deutsch als Muttersprache sowie eine beidseitige Normalhörigkeit gemäß DIN EN ISO 8253–1 12 . Vor Beginn der Messungen wurden das Geschlecht und das Alter der Probanden dokumentiert und eine Aufklärung über den allgemeinen Ablauf sowie die durchzuführenden Untersuchungen vorgenommen. Es folgte eine Anamnese gemäß DIN, welche Fragen zur Lärmexposition in der Vergangenheit, zur Einnahme von ototoxischen Medikamenten und zu bekannten Erkrankungen der Ohren beinhaltete. Sodann wurde das Hörvermögen im nach DIN kalibrierten Reintonaudiogramm (Madsen Astera, Fa. Otometrics) mithilfe von Kopfhörern festgestellt und beurteilt, um eine Normalhörigkeit sicherzustellen 12 13 14 . Diese besteht nach DIN EN ISO 8253–1, sofern die Reintonhörschwellen über die Luftleitung im Frequenzbereich von 125–8000Hz einen maximalen Hörverlust von 10dB HL in maximal 2 Frequenzen aufweisen. Nachfolgend schlossen sich die Untersuchungen des Sprachverstehens im Störschall an, wobei, wie in der Voruntersuchung von *Memmeler et al* ., der OLSA-S und der aFBE-S vergleichend zur Anwendung kamen 5 . Für eine Ausgewogenheit im Testablauf und eine höhere Kohärenz der Ergebnisse begannen je 20 Probanden randomisiert mit einem der beiden Verfahren. Die Gruppe aFBE-S 1 bzw. OLSA-S 1 bestand aus Probanden, bei denen der aFBE-S bzw. der OLSA-S als erstes Verfahren angewendet wurde, während bei der Gruppe aFBE-S 2 bzw. OLSA-S 2 der aFBE-S bzw. der OLSA-S als zweites Verfahren zum Einsatz kamen.

Die Präsentation des Stör- und Nutzsignals wurde binaural im Freifeld durchgeführt, jeweils aus einem Winkel von 0° (S _0_ N _0_ -Situation). Vor Beginn der Untersuchungen erhielten die Probanden die Anweisung, eine aufrechte Sitzposition einzunehmen und den Kopf während der Messungen weder zu drehen noch sich den Lautsprechern zu nähern. Hierdurch wurde ein Abstand von 1 Meter zwischen Lautsprecher und der Kopfmitte eingehalten. Sowohl die Auswahl der Testlisten als auch deren Reihenfolge waren für beide Sprachverstehenstests zufällig. Die gesamte Versuchsdurchführung fand in einem nach DIN EN ISO 8253–3 genormten akustischen Raum der BAGUS GmbH in Essen statt 13 .

### Adaptive Messung des Freiburger Einsilbertests im Störschall

Der FBE wurde in digitalisierter Form – unter Verwendung einer im Jahr 1969 veröffentlichten und nach DIN 45621–1 genormten Aufnahme – durchgeführt 4 . Zusätzlich zum Sprachsignal kam ein digitalisiertes Störgeräusch zum Einsatz, das CCITT-Rauschen 15 . Es wurde vor Durchführung der Messung auf einen Pegel von 65dB SPL eingestellt und kontinuierlich präsentiert. Für die adaptive Anwendung erhielt der Freiburger Einsilbertest den Algorithmus des adaptiv messenden Oldenburger Satztests, sodass der Sprachschalldruckpegel in Relation zur Anzahl der verstandenen Wörter verändert wurde 16 .

Der Algorithmus des Oldenburger Satztests sieht für die Bestimmung der 50%-SVS vor, den Pegel bei ≤2 von 5 richtig wiedergegebenen Wörtern pro Satz zu erhöhen bzw. bei ≥3 von 5 zu senken ( Tab. 1 ). Dabei erfolgt die Pegelanpassung innerhalb der ersten 5 Sätze in größeren Schritten 16 . Analog zu den aus 5 Wörtern bestehenden Sätzen des OLSAs wurden die Einsilber des FBE in Gruppen zu jeweils 5 Wörtern eingeteilt. Somit bestand jede Liste aus 4 5er-Gruppen, was 4 Pegeländerungen pro Testliste bedeutete. Die verstandenen Wörter wurden manuell durch den Versuchsleiter als richtig oder falsch markiert. Erst danach erfolgte die Präsentation des darauffolgenden Wortes. Wie beim Störschall wurde bei der Präsentation der Einsilber zu Beginn ein Pegel von 65dB SPL eingestellt, der sich im Verlauf der Messung jedoch veränderte. Hierfür wurde nach Präsentation einer 5er-Gruppe die Anzahl der korrekt verstandenen Wörter in einer vorgefertigten Excel-Tabelle eingetragen und die adaptive Pegeländerung gemäß dem Algorithmus des OLSA automatisch berechnet. Anschließend folgte die manuelle Adjustierung des Sprachpegels für die Darbietung der darauffolgenden 5er-Gruppe in der Anwendungssoftware des FBE durch den Versuchsleiter.

**Table:** **Tab. 1** Adaptive Pegeländerung für die manuelle Durchführung des Oldenburger Satztests, modifiziert nach „Oldenburger Satztest Bedienungsanleitung für den manuellen Test auf Audio CD“ 16 .

	Pegeländerung der Sprache	
richtig verstandene Wörter im vorangegangenen Satz	Sätze 2–5	Sätze 6–31
5	–3dB	–2dB
4	–2dB	–1dB
3	–1dB	0dB
2	+1dB	0dB
1	+2dB	+1dB
0	+3dB	+2dB

Für die Datenerfassung kamen somit 2 Computer zum Einsatz. Dabei diente ein PC zur Ausführung der Software des FBE, also zur Durchführung der Sprachaudiometrie. Der zweite PC berechnete die Pegeländerungen dem Algorithmus nach mithilfe einer Microsoft-Excel-Tabelle, die die ermittelten Daten festhielt. Insgesamt erhielt jeder Proband 5 Testlisten mit jeweils 4 5er-Gruppen, sodass sich der Sprachpegel bis zu 20-mal verändern konnte. Neben der Erfassung der Sprachverständlichkeit erfolgte eine Dokumentation der erforderlichen Zeit. Dafür wurde am PC, in dem die Daten in der Excel Tabelle festgehalten wurden, eine Stoppuhr verwendet und die Zeit nach 3, 4 und schließlich 5 Testlisten gemessen und notiert. Die ohne Anwendung von Störschall als nicht perzeptiv äquivalent kritisierten Listen 5, 11, 12 und 15 wurden – analog zu *Memmeler et al.* (2018, 2024) – ausgeschlossen 5 11 .

### Durchführung des Oldenburger Satztests im Störschall

Jeder Proband bekam für die Durchführung des OLSA-S zunächst eine zufällig ausgewählte Trainingsliste präsentiert, die möglichen Habituations- und Lerneffekten vorbeugen sollte und sich aus 30 Sätzen zu je 5 Wörtern zusammensetzte. Darauf wurde eine weitere, zufällig ausgewählte Testliste vorgelegt, die sich ebenfalls aus 30 Sätzen zusammensetzte und der Berechnung der Signal-Rausch-Abstände für das 50%- Sprachverstehen (S/N50) diente. Das Störgeräusch wurde fortlaufend mit einem Pegel von 65dB SPL eingesetzt, während sich der Sprachpegel gemäß dem Algorithmus der adaptiven Pegeländerung im Testverlauf veränderte 16 . Die Probanden erhielten die Anweisung, nach der Präsentation des Testsatzes die verstandenen Inhalte laut und deutlich zu wiederholen. Eine deutliche Artikulation war wichtig, da der Versuchsleiter per Anwendungssoftware des OLSA-S aus einem Auswahlfeld die richtig nachgesprochenen Inhalte manuell markieren musste. So wurde der Sprachpegel des darauffolgenden Satzes adaptiv, je nach der Anzahl der richtig verstandenen Wörter, angepasst. Sowohl die Änderungen des Sprachpegels als auch die Berechnungen des S/N50 wurden selbstständig von der Software des OLSA-S berechnet und angepasst.

### Statistische Analyse

Die Daten wurden mithilfe von Microsoft Excel-Tabellen erfasst und auf die Software IBM SPSS 29.0.1.0 für die statistische Auswertung übertragen. Weiterhin diente das Programm Graph Pad Prism 10.0 zur statistischen Darstellung der Ergebnisse. Hierfür wurden Streudiagramme der jeweiligen Mittelwerte des aFBE-S 3, 4, 5 und des OLSA-S mit Standardabweichungen abgebildet. Die Überprüfung der Normalverteilung erfolgte mittels Shapiro-Wilk-Tests, der sowohl für den aFBE-S 3, 4, 5 als auch für den OLSA-S keine Normalverteilung ergab. Deshalb wurde auch für den Vergleich der S/N50 zwischen dem aFBE-S 3, 4 und 5 und dem OLSA-S als abhängige Stichprobe der nichtparametrische Vorzeichentest durchgeführt. Zusätzlich erfolgte eine Gegenüberstellung der S/N50 des aFBE-S mit 3, 4 und 5 Testlisten mit dem Friedman-Test. Um die Zusammenhänge der ermittelten SNR zwischen dem aFBE-S 3, 4 und 5 und dem OLSA-S zu ergründen, wurde jeweils der Pearson-Korrelationskoeffizient berechnet. Weiterhin wurde für den Gruppenvergleich der Geschlechter sowie den Einfluss der Präsentationsreihenfolge auf die Signal-Rausch-Abstände der Mann-Whitney-U-Test mit jeweils 2 unabhängigen Stichproben ausgeführt. Zur Gegenüberstellung und statistischen Analyse des Zeitaufwands beider Verfahren kam angesichts normalverteilter Daten der gepaarte t-Test zur Anwendung. Als Signifikanzniveau galt in allen Berechnungen ein p-Wert von ≤0,05.

Aufgrund der erhöhten Pegelfluktuationen der Einsilber wurde in dieser Untersuchung dem Vorschlag von *Holube et al.* gefolgt, die Anpassung des Sprachsignals nach dem äquivalenten Dauerschalldruckpegel der Sprache (L _eq_ ) vorzunehmen. Gleichzeitig erfolgte eine Berücksichtigung der verringerten Verdeckungseigenschaften des CCITT-Rauschens gegenüber dem sprachsimulierenden Rauschen des OLSA-S, sodass die ermittelten Signal-Rausch-Abstände für den aFBE-S nachträglich um –5,9dB nach unten angepasst wurden 10 .

## Ergebnisse

### Vergleich der Signal-Rausch-Abstände

**Abb. 1:**
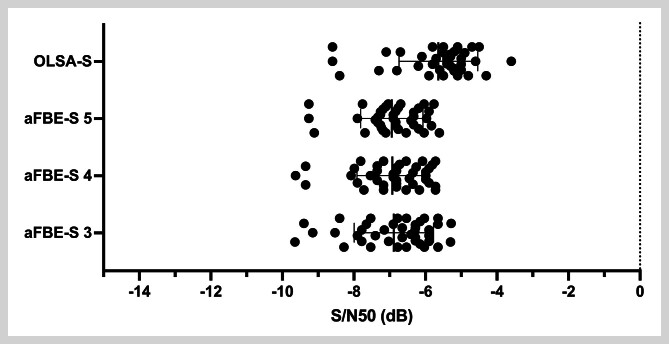
Vergleich der ermittelten, vom Geschlecht unabhängigen Signal-Rausch-Abstände (S/N50 [dB]) zwischen dem adaptiven Freiburger Einsilbertest mit 3, 4 und 5 Testlisten (aFBE-S 3, aFBE-S 4, aFBE-S 5) und dem Oldenburger Satztest (OLSA-S) im Störschall.

Die S/N50 erstreckten sich beim OLSA-S zwischen –3,6dB und –8,6dB. Demgegenüber betrugen die Werte der S/N50 beim aFBE-S 3 zwischen –5,28dB und –9,65dB, beim aFBE-S 4 zwischen –5,72 dB und –9,63 dB sowie beim aFBE-S 5 Testlisten zwischen –5,61dB und –9,26dB. Die vom Geschlecht unabhängigen mittleren S/N50 fielen dabei beim aFBE-S 3 mit –6,88±1,11dB, beim aFBE-S 4 mit –6,92±0,99dB und beim aFBE-S 5 mit –6,94±0,87dB größer aus als beim OLSA-S mit –5,64±1,10dB. Es konnte ein signifikanter Unterschied zwischen den S/N50 des OLSA-S und denen des aFBE-S 3 (p<0,001), des aFBE-S 4 (p<0,001) und des aFBE-S 5 (p<0,001) festgestellt werden ( Tab. 2 , Abb. 1 ).

**Table:** **Tab. 2** Vergleich der vom Geschlecht unabhängigen Signal-Rausch-Abstände (S/N50) in Bezug auf das Minimum (Min.), das Maximum (Max.), die Mittelwerte und die 95%-Konfidenzintervalle (95%-KI).

	S/N50			
	Min.	Max.	Mittelwert	95%-KI
OLSA-S	–3,6dB	–8,6dB	–5,64dB±1,10dB	–0,35–0,31
aFBE-S 3	–5,28dB	–9,65dB	–6,88dB±1,11dB	–0,34–0,33
aFBE-S 4	–5,72dB	–9,63dB	–6,92dB±0,99dB	–0,33–0,30
aFBE-S 5	–5,61dB	–9,26dB	–6,94dB±0,87dB	–0,29–0,27

### Korrelation der Signal-Rausch-Abstände

Zwischen den Ergebnissen des OLSA-S und denen des aFBE-S 3 bestand bei einem Korrelationskoeffizienten von r=0,386 (p=0,014) ein moderat positiv linearer Zusammenhang. Ähnliche Korrelationen wurden auch zwischen den Signal-Rausch-Abständen des OLSA-S und denen des aFBE-S 4 und aFBE-S 5 festgestellt. Demnach lag der Korrelationskoeffizient bei r=0,460 (p=0,003) zwischen dem OLSA-S und dem aFBE-S 4 sowie bei r=0,474 (p=0,002) zwischen dem OLSA-S und dem aFBE-S 5, was einer moderaten Korrelation entspricht.

### Einfluss der Präsentationsreihenfolge und Lerneffekte

Beim aFBE-S belief sich der mittlere S/N50 mit 3 Testlisten auf –6,77±1,09dB (KI –0,55–0,41) in der Gruppe aFBE-S 1 und auf –6,99±1,15dB (KI –0,47–0,50) in der Gruppe aFBE-S 2, mit 4 Testlisten auf –6,83±0,99dB (KI –0,50–0,37) in der Gruppe aFBE-S 1 und auf –7,02±1,01dB (KI –0,45–0,44) in der Gruppe aFBE-S 2 sowie mit 5 Testlisten auf –6,87±0,90dB (KI –0,45–0,36) in der Gruppe aFBE-S 1 und –7,01±0,86dB (KI –0,39–0,37) in der Gruppe aFBE-S 2. In Bezug auf die Versuchsreihenfolge konnten beim aFBE-S mit 3, 4 und 5 Testlisten keine signifikanten Unterschiede zwischen den Werten der S/N50 festgestellt werden (3 Testlisten: p=0,626; 4 Testlisten: p=0,379; 5 Testlisten: p=0,279). Beim OLSA ergab der mittlere S/N50 in der Gruppe OLSA-S 1 –5,82±1,17dB (KI –0,56–0,48) und in der Gruppe OLSA-S 2 –5,47±1,03dB (KI –0,53–0,40). Die ermittelten Werte der S/N50 wiesen zwischen beiden Gruppen keine signifikanten Unterschiede (p=0,694) auf ( Tab. 3 ).

**Table:** **Tab. 3** Vergleich der gemittelten, vom Geschlecht unabhängigen Signal-Rausch-Abstände (S/N50) des OLSA-S und des aFBE-S 3, 4 und 5 in Bezug auf die Präsentationsreihenfolge sowie deren jeweilige Signifikanz (Sig.) zueinander.

	S/N50		
	1. Verfahren	2. Verfahren	Sig. (2-seitig)
OLSA-S	–5,82±1,17dB	–5,47±1,03dB	p=0,694
aFBE-S 3	–6,77±1,09dB	–6,99±1,15dB	p=0,626
aFBE-S 4	–6,83±0,99dB	–7,02±1,01dB	p=0,379
aFBE-S 5	–6,87±0,90dB	–7,01±0,86dB	p=0,279

Hinsichtlich der Lerneffekte des OLSA-S konnten signifikante Unterschiede in den mittleren S/N50 zwischen der Trainingsliste und der darauffolgenden Testliste festgestellt werden. Im Durchschnitt erzielten die Probanden bei der Trainingsliste eine um 1,6dB geringere S/N50 als bei der Testliste (p<0,001; KI –1,60–0,71). Beim aFBE-S wurde keine Trainingsliste verwendet, allerdings konnten auch keine signifikanten Unterschiede zwischen der Eingewöhnungsphase und der eigentlichen Testphase beobachtet werden. Die durchschnittliche Erhöhung der S/N50 in der Eingewöhnungsphase ergab 0,3dB beim aFBE-S 3 (p=0,148; KI –0,74–1,36), 0,28dB beim aFBE-S 4 (p=0,108; KI –0,69–0,13) und 0,19dB beim aFBE-S 5 (p=0,150; KI –0,61–0,12).

### Geschlechtsspezifische Unterschiede

Um geschlechtsspezifische Unterschiede der S/N50 analysieren zu können, wurden die Ergebnisse von 2 Gruppen, die jeweils aus ausschließlich männlichen bzw. ausschließlich weiblichen Probanden bestanden, untersucht. Die Gruppe der weiblichen Probanden wies dabei einen mittleren S/N50 von -6,91±1,03dB (KI -0,43–0,42) bei 3 Testlisten (aFBE-S 3) auf sowie von –7,02±0,91dB (KI –0,38–0,35) bei 4 Testlisten (aFBE-S 4) und von –7,05±0,75dB (KI –0,31–0,30) bei 5 Testlisten (aFBE-S 5). Die männlichen Probanden hingegen zeigten einen mittleren S/N50 von –6,85±1,23dB (KI -0,54–0,52) bei 3 Testlisten, von –6,81±1,10dB (KI -0,50–0,45) bei 4 Testlisten und von –6,81±1,01dB (KI -0,47–0,42) bei 5 Testlisten. Es wurden keine signifikanten, geschlechtsspezifischen Unterschiede beim aFBE-S mit 3, 4 und 5 Testlisten festgestellt (aFBE-S 3: p=0,683, aFBE-S 4: p=0,236, aFBE-S 5: p=0,165). Hinsichtlich geschlechtsspezifischer Unterschiede beim OLSA-S zeigten die weiblichen Probanden einen mittleren S/N50 von –5,65±1,14dB (KI –0,54–0,42), während die männlichen Probanden einen vergleichbaren mittleren S/N50 von –5,62±1,08dB (KI –0,46–0,47) aufwiesen. Es konnten ebenfalls keine signifikanten Unterschiede (p=0,723) zwischen beiden Geschlechtern festgestellt werden ( Tab. 4 ).

**Table:** **Tab. 4** Geschlechtsspezifische Unterschiede des OLSA-S und des aFBE-S 3, 4 und 5 in Bezug auf die mittleren Signal-Rausch-Abstände (S/N50) und deren Signifikanz (Sig.) zueinander.

	S/N50		
	weiblich	männlich	Sig. (2-seitig)
OLSA-S	–5,65±1,14dB	–5,62±1,08dB	p=0,723
aFBE-S 3	–6,91±1,03dB	–6,85±1,23dB	p=0,683
aFBE-S 4	–7,02±0,91dB	–6,81±1,10dB	p=0,236
aFBE-S 5	–7,05±0,75dB	–6,81±1,01dB	p=0,165

### Zeitaufwand

Der zeitliche Aufwand wurde für die Durchführung des aFBE-S mit 3, 4 und 5 Testlisten und des OLSA-S samt Trainingsliste erfasst. Die durchschnittliche Testdauer beim aFBE-S betrug 323±24s (5min 23 s±24 s) mit 3 Testlisten, 424±32s (7min 04s±32s) mit 4 Testlisten sowie 523±34s (8min 43s±34s) mit 5 Testlisten. Der dafür erforderliche Zeitaufwand reichte beim aFBE-S mit 3 Testlisten von 280–376s, beim aFBE-S mit 4 Testlisten von 373–496s und beim aFBE-S mit 5 Testlisten von 469–582s für die kürzeste und die längste Messung.

**Abb. 2:**
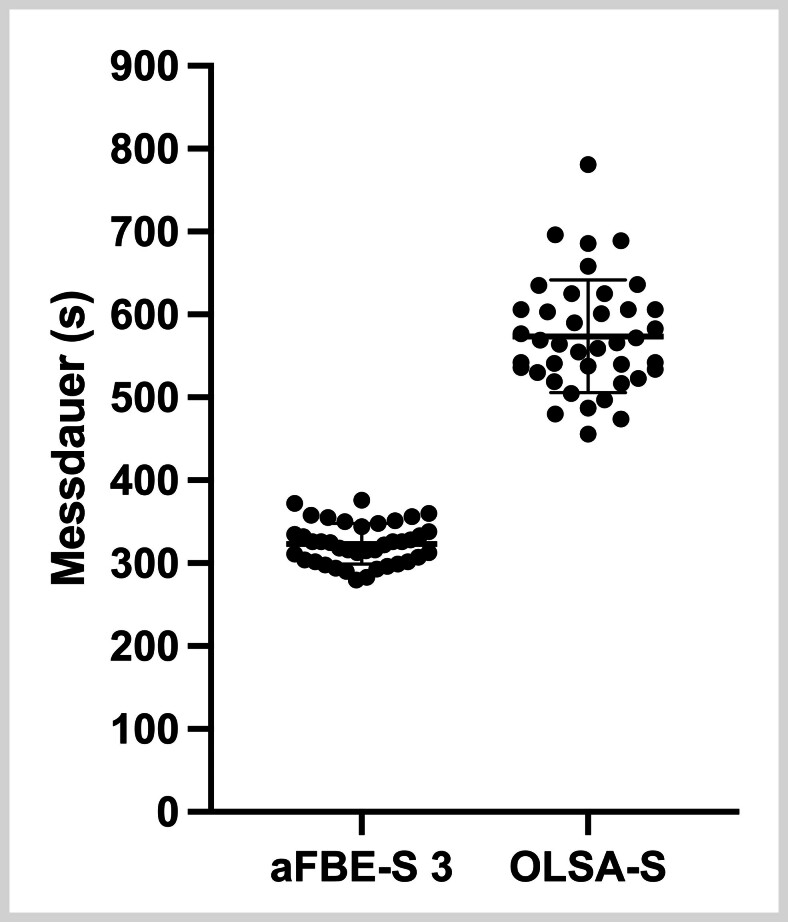
Vergleich des jeweiligen Zeitaufwands in Sekunden (s) zwischen dem adaptiven Freiburger Einsilbertest mit 3 Testlisten (aFBE-S 3) und dem Oldenburger Satztest im Störschall (OLSA-S).

**Abb. 3:**
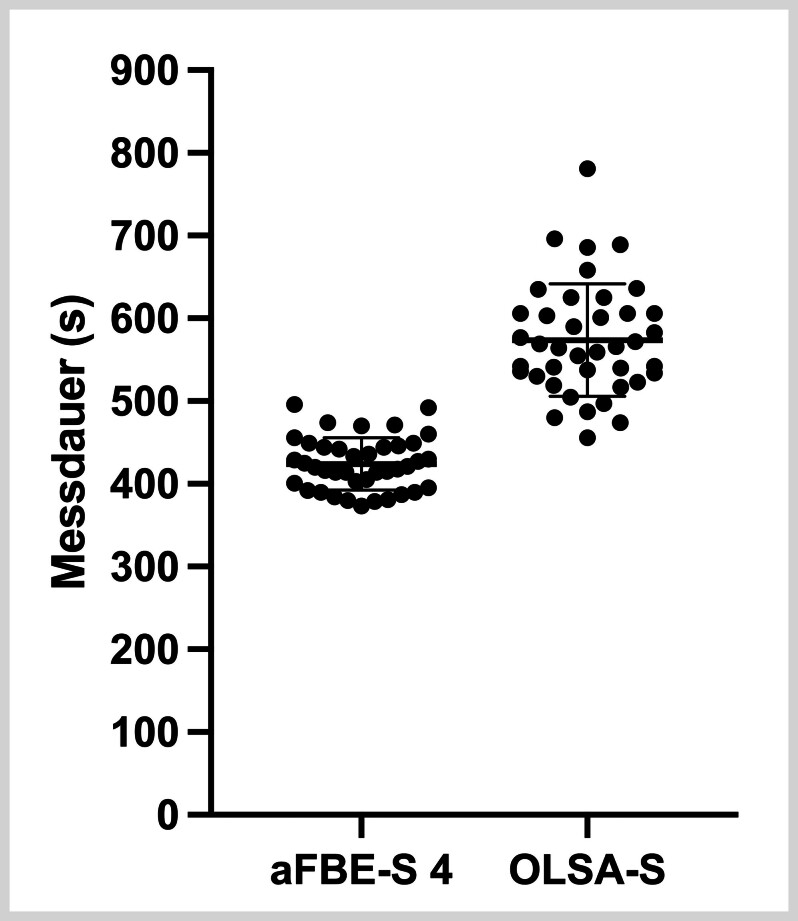
Vergleich des jeweiligen Zeitaufwands in Sekunden (s) zwischen dem adaptiven Freiburger Einsilbertest mit 4 Testlisten (aFBE-S 4) und dem Oldenburger Satztest im Störschall (OLSA-S).

**Abb. 4:**
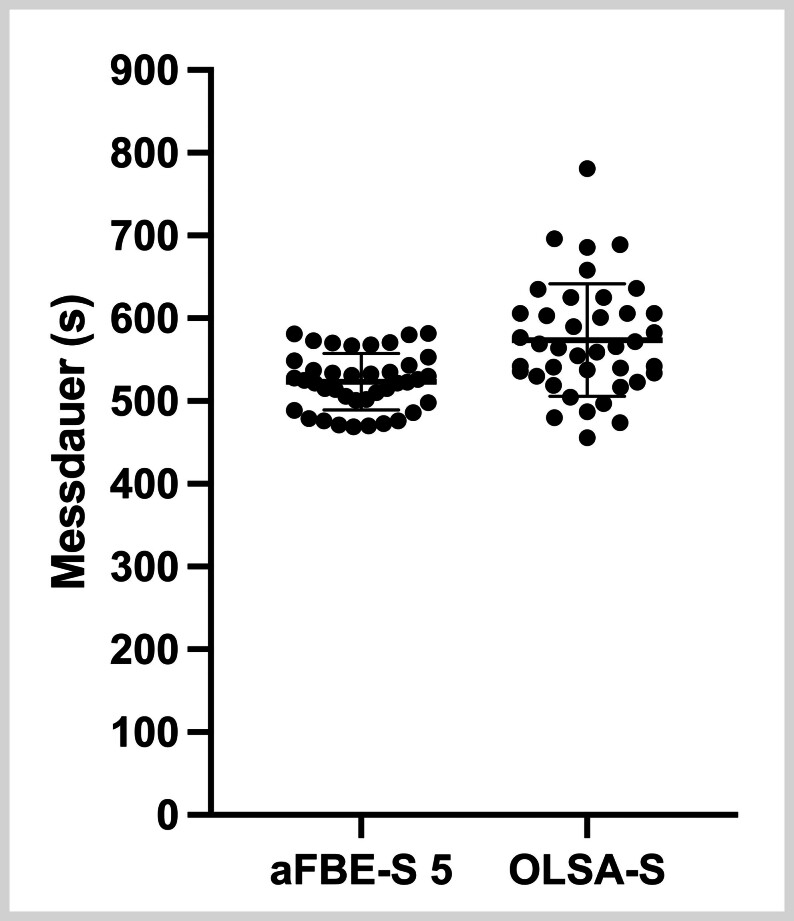
Vergleich des jeweiligen Zeitaufwands in Sekunden (s) zwischen dem adaptiven Freiburger Einsilbertest mit 5 Testlisten (aFBE-S 5) und dem Oldenburger Satztest im Störschall (OLSA-S).

Hingegen nahm der OLSA-S im Durchschnitt 574±68s (9min 34s±68s) in Anspruch, wobei die Testdauer zwischen 456s (7min 36s) für die kürzeste und 781 s (13min 01s) für die längste Messung variierte ( Tab. 5 , Abb. 2 , Abb. 3 , Abb. 4 ).

**Table:** **Tab. 5** Geschlechtsunabhängiger mittlerer Zeitaufwand des OLSA-S und des aFBE-S 3, 4 und 5.

	Zeitaufwand	
	Mittelwert in Sekunden (s)	95%-Konfidenzintervall
OLSA-S	574±68s	–19,4–20,5s
aFBE-S 3	323±24s	–7,5–7,5s
aFBE-S 4	424±32s	–9,5–9,1s
aFBE-S 5	523±34s	–9,9–10s

Hinsichtlich des durchschnittlichen Zeitbedarfs ließ sich der aFBE-S mit 3, 4 und 5 Testlisten somit schneller als der OLSA-S durchführen, wobei die Unterschiede signifikant waren (OLSA-S/aFBE-S 3: p<0,001; OLSA-S/aFBE-S 4: p<0,001; OLSA-S/aFBE-S 5: p<0,001). Die Nullhypothese 3 kann somit verworfen und die Alternativhypothese 3 angenommen werden.

## Diskussion

Mit dieser Arbeit sollten weiterführende Erkenntnisse über die Umsetzbarkeit der adaptiven Sprachpegeländerung am FBE im Störgeräusch (aFBE-S) gewonnen und die Durchführung in einer verkürzter Testversion mittels 3, 4 bzw. 5 Testlisten evaluiert werden. Gleichzeitig erfolgte eine Gegenüberstellung der ermittelten Ergebnisse mit dem adaptiv messenden Oldenburger Satztest, der ebenfalls im Störgeräusch angewendet wurde (OLSA-S). Untersucht wurden die Parameter des Signal-Rausch-Abstandes und des Zeitaufwandes.

### Signal-Rausch-Abstände

Hinsichtlich der geschlechtsunabhängigen SNR stellten sich signifikante Unterschiede zwischen dem OLSA-S und dem aFBE-S heraus. Die mittlere SNR fiel für die 50%-SVS beim aFBE-S mit 3, mit 4 und 5 Testlisten betragsmäßig größer aus als beim OLSA. Hingegen bestanden zwischen den Signal-Rausch-Abständen des aFBE-S 3, des aFBE-S 4 und des aFBE-S 5 keine signifikanten Unterschiede (p=0,885). Dies stimmt mit der vorhandenen Literatur überein und lässt annehmen, dass der aFBE-S 3 für den Erhalt valider Ergebnisse bereits hinreichend geeignet ist und empfohlen werden kann 11 17 . Als eine Erklärung für den in dieser Untersuchung festgestellten geringeren SNR beim OLSA-S im Vergleich zum aFBE-S könnten die überwiegend sinnfreien Sätze im Sprachmaterial genannt werden, die über eine geringe Redundanz verfügen 18 . Zusätzlich erfordert die Durchführung des OLSA, im Gegensatz zum FBE, das Hörverstehen von ganzen Sätzen. Hierdurch werden kognitive Ressourcen, insbesondere für die Zwischenspeicherung und Verarbeitung der auditiven Informationen, im höheren Maße beansprucht 19 20 . Es ist anzunehmen, dass sich dies auch in den Standardabweichungen der SNR-Werte widerspiegelt, die beim OLSA-S mit ±1,10dB und beim aFBE-S 3 mit ±1,11dB höher ausfielen als beim aFBE-S 4 mit ±0,99dB und beim aFBE-S 5 mit ±0,87dB. Zusätzlich ist zu beobachten, dass die Streuung der SNR-Werte beim aFBE-S mit zunehmender Anzahl an Testlisten abnimmt, was mit einer erhöhten Reproduzierbarkeit bzw. Genauigkeit der Ergebnisse bei zunehmender Testlistenanzahl einhergeht.

Letztlich ist anzumerken, dass auch die Einsilber des FBE eine begrenzte Redundanz und eine im Vergleich zum OLSA flachere Diskriminationsfunktion aufweisen 1 20 . Die Ursachen für die Unterschiede der SNR sind deshalb vermutlich vielfältig und nicht ausschließlich auf das Sprachmaterial zurückzuführen.

So können nichtaudiometrische, interindividuelle Unterschiede, wie z.B. die Motivation oder der allgemeine Wortschatz der Probanden, erheblichen Einfluss auf das Ergebnis haben 21 22 . Dies betrifft auch die Sprachkompetenz, welche sich in dieser Untersuchung möglicherweise anhand der größeren Varianz bei der Testdauer des OLSA-S zeigte und nahelegen könnte, dass die Wiedergabe der ganzen Sätze des OLSA zu einer individuell variableren, kognitiven Beanspruchung der Probanden führt als die Einsilber des FBE 19 . In dieser Untersuchung setzte sich das Probandenkollektiv relativ ausgewogen aus Studierenden und Auszubildenden zusammen.

### Korrelation der Signal-Rausch-Abstände

Die Untersuchung der linearen Beziehungen der SNR zwischen dem OLSA-S und dem aFBE-S ergab eine moderat positive Korrelation. In früheren Studien konnten bereits signifikante Zusammenhänge zwischen den Ergebnissen des FBE und denen weiterer Sprachverständlichkeitstests, wie beispielsweise dem OLSA, ermittelt werden 23 24 . Basierend auf diesen Ergebnissen wurde geschlussfolgert, dass der Freiburger Sprachtest zukünftig durch den OLSA-S ersetzt werden könnte 23 . Andersherum könnte man nach den Ergebnissen dieser Studie überlegen, ob nicht vielmehr der Freiburger Sprachtest den OLSA ersetzen könnte, da der FBE bereits flächendeckend zum Einsatz kommt und wahrscheinlich auch in einer kommerziell erhältlichen adaptiven Form kostengünstiger und schneller durchführbar wäre. Die grundsätzlichen Unterschiede zwischen einem Satz- und Einsilbertest bleiben jedoch weiterhin bestehen. Außerdem ist zu erwähnen, dass es bezüglich der Korrelation zwischen dem OLSA-S und dem aFBE-S bisher an kumulativen Daten mangelt bzw. dass die vorhandenen Ergebnisse denen dieser Studie widersprechen. So haben z.B. *Memmeler et al.* (2018) mit r=-0,03 (p=0,87) keine Korrelation zwischen dem OLSA-S und dem aFBE-S mit 7,5 Testlisten ermitteln können 5 . Es wäre daher für die Übertragung der Ergebnisse vom aFBE-S auf den OLSA-S bzw. vom OLSA-S auf den aFBE-S notwendig, weitere fundierte Daten zur Korrelation der Signal-Rausch-Abstände zwischen beiden Verfahren zu erheben. Dies wäre vor allem für die Begutachtung von großer Relevanz.

### Einfluss von Versuchsreihenfolge und Geschlechterverteilung auf die Signal-Rausch-Abstände

Als mögliche Erklärung für den in dieser Studie fehlenden Einfluss der Versuchsreihenfolge auf das SNR könnte die durch die Probanden selbstbestimmte Pause von bis zu 30 Minuten zwischen den beiden Verfahren genannt werden. Dadurch sollte die Höranstrengung, also die Beanspruchung kognitiver Ressourcen im Sprachverstehen, berücksichtigt und deren Einfluss auf das Testergebnis reduziert werden 25 26 . Ferner wurde gemäß den Empfehlungen für den OLSA-S eine Trainingsliste hinzugezogen, um mögliche Verzerrungen der Ergebnisse durch prozedurale Lerneffekte minimieren zu können 27 . Der Lerneffekt in dieser Studie belief sich dabei im Durchschnitt auf 1,16dB, womit sich der SNR zwischen der 1. und der 2. Testliste um 1,16dB vergrößerte. Außerdem fanden beim OLSA-S lediglich die letzten 19 der insgesamt 30 Sätze für die Berechnung der 50%-SVS Berücksichtigung, sodass der Einfluss von Ungenauigkeiten zu Beginn der Messung auf das Testergebnis weitgehend reduziert werden konnte. In der Gegenüberstellung wurde beim aFBE-S, bedingt durch die widersprüchlichen Meinungen in der einschlägigen Literatur, auf die Anwendung einer Trainingsliste verzichtet 28 29 . Damit bestand zwar keine Eingewöhnung im Rahmen einer Versuchssimulation, allerdings wurden – analog zum Vorgehen des OLSA-S – ausschließlich die letzten 64,5% aller Sprachpegel mit in die Berechnung der 50%- SVS einbezogen bzw. die ersten 35,5% des Sprachmaterials ausgeschlossen. Es stellte sich in dieser Untersuchung heraus, dass selbst ohne die Anwendung einer entsprechenden Eingewöhnungsphase mit einer Differenz von bis zu 0,3dB keine signifikanten Unterschiede der gemittelten SNR beim aFBE-S bestehen. Damit erübrigt sich theoretisch die Eingewöhnungsphase für den aFBE-S.

### Zeitaufwand

In dieser Untersuchung war der durchschnittliche Zeitaufwand für den OLSA-S signifikant größer als für den aFBE-S 3, 4 und 5. Als wesentliche Ursache hierfür könnte die beim OLSA-S verwendete Trainingsliste genannt werden, auf die beim aFBE-S aufgrund der uneindeutigen Empfehlungen verzichtet wurde 28 29 . Des Weiteren ließe sich anführen, dass der aFBE-S in seiner Version verkürzt wurde. Somit standen ohne Berücksichtigung der Trainingslisten maximal 100 Einsilber beim aFBE-S den 150 Wörtern des OLSA-S gegenüber. Folglich konnte der aFBE-S insgesamt deutlich schneller durchgeführt werden, obwohl die durchschnittliche Zeit pro Liste die in der Literatur angegebene Referenz von 1,5 Minuten überschritt und keine automatische Software für die Berechnung der Pegeländerung zur Verfügung stand, welche den Zeitbedarf noch weiter hätte verringern können 30 . Gleichzeitig wurde jedoch auch für den OLSA-S mehr Zeit benötigt als die in der Literatur angegebene Referenz von 4 Minuten pro Liste, wobei mit ±68 Sekunden eine im Vergleich zum aFBE-S deutlich größere Streuung auftrat 27 . Somit sind auch die individuellen Unterschiede der Probanden im Antwortverhalten zu berücksichtigen, welche den Zeitbedarf maßgeblich beeinflussten.

Ein möglicher Ansatz, die Testdauer des OLSA-S und damit auch die kognitiven Belastungen reduzieren zu können, wurde kürzlich von *Schmid et al.* untersucht. Hierbei erfolgte mithilfe der Bayes’schen Statistik eine kontinuierliche Analyse verschiedener Parameter der psychometrischen Funktion. Dadurch wurde neben einer individuellen Anpassung des Testverfahrens an die Hörfähigkeiten der Patienten auch über Wahrscheinlichkeitsberechnungen eine im Durchschnitt 1,3 Minuten kürzere Testdauer als die ursprüngliche adaptive Methode erreicht 31 . Es wäre zu überprüfen, ob diese bisher generierten Ergebnisse reproduzierbar sind und ob sich dieser Ansatz eventuell auch auf den FBE-S übertragen lässt.

## Fazit für die Praxis

- Die verkürzte Version des aFBE-S mit 3, 4 und 5 Testlisten liefert im Vergleich zum OLSA-S signifikant größere Signal-Rausch-Abstände (SNR).
- Zwischen den SNR des aFBE-S mit 3, 4 und 5 Testlisten bestehen hingegen keine signifikanten Unterschiede, sodass der aFBE-S 3 zur Bestimmung der 50%-SVS ausreichend ist und für die klinische Praxis empfohlen werden kann.
- Der aFBE-S mit 3 Testlisten lässt sich signifikant schneller durchführen als der OLSA-S und weist diesbezüglich eine geringere Streuung auf.
- Sowohl für den aFBE-S als auch für den OLSA-S besteht kein Einfluss der Versuchsreihenfolge und des Geschlechts auf die Ergebnisse der SNR.
- Für eine effiziente Anwendung des aFBE-S im klinischen Gebrauch wäre eine automatisierte Software zu empfehlen.
